# 123VCF: an intuitive and efficient tool for filtering VCF files

**DOI:** 10.1186/s12859-024-05661-5

**Published:** 2024-02-14

**Authors:** Milad Eidi, Samaneh Abdolalizadeh, Soheila Moeini, Masoud Garshasbi, Javad Zahiri

**Affiliations:** 1 Department of Medical Genetics, Faculty of Medical Sciences, Tarbiat Modares University, Tehran, Iran; 2 Department of Genetics and Molecular Medicine, School of Medicine, Zanjan University of Medical Sciences (ZUMS), Zanjan, Iran; 3 Département de Biochimie et Médecine Moléculaire, Université de Montréal, Montreal, QC Canada; 4 Research Centre, Montreal Heart Institute, Montreal, QC Canada; 5 Department of Neuroscience, University of California San Diego, San Diego, CA USA

**Keywords:** Next generation sequencing, VCF, VCF filtering, Variant analysis, Variant filtering, Exome sequencing, Genome sequencing

## Abstract

**Background:**

The advent of Next-Generation Sequencing (NGS) has catalyzed a paradigm shift in medical genetics, enabling the identification of disease-associated variants. However, the vast quantum of data produced by NGS necessitates a robust and dependable mechanism for filtering irrelevant variants. Annotation-based variant filtering, a pivotal step in this process, demands a profound understanding of the case-specific conditions and the relevant annotation instruments. To tackle this complex task, we sought to design an accessible, efficient and more importantly easy to understand variant filtering tool.

**Results:**

Our efforts culminated in the creation of 123VCF, a tool capable of processing both compressed and uncompressed Variant Calling Format (VCF) files. Built on a Java framework, the tool employs a disk-streaming real-time filtering algorithm, allowing it to manage sizable variant files on conventional desktop computers. 123VCF filters input variants in accordance with a predefined filter sequence applied to the input variants. Users are provided the flexibility to define various filtering parameters, such as quality, coverage depth, and variant frequency within the populations. Additionally, 123VCF accommodates user-defined filters tailored to specific case requirements, affording users enhanced control over the filtering process. We evaluated the performance of 123VCF by analyzing different types of variant files and comparing its runtimes to the most similar algorithms like BCFtools filter and GATK VariantFiltration. The results indicated that 123VCF performs relatively well. The tool's intuitive interface and potential for reproducibility make it a valuable asset for both researchers and clinicians.

**Conclusion:**

The 123VCF filtering tool provides an effective, dependable approach for filtering variants in both research and clinical settings. As an open-source tool available at https://project123vcf.sourceforge.io, it is accessible to the global scientific and clinical community, paving the way for the discovery of disease-causing variants and facilitating the advancement of personalized medicine.

## Background

The advent of next-generation sequencing (NGS) technologies has revolutionized the field of genomics, enabling the analysis of large-scale genomic datasets with unprecedented accuracy and resolution. However, the sheer volume of data generated by NGS requires efficient and reliable tools for variant analysis. This analysis typically involves the identification of disease-causing variants by filtering out irrelevant variants using annotation-based filtering, a critical step in the analysis pipeline that requires an understanding of both the case's conditions and available annotations [1, 2].

Several standalone and web-based tools, such as ANNOVAR, wANNOVAR, VEP, and SnpEff, are available to annotate variants [3–6]. However, variant filtration, the subsequent step in the analysis pipeline, requires specialized, flexible, and user-friendly tools. Graphical User Interface (GUI) based tools, such as VCF.Filter, VCF-Miner, and BrowseVCF, enable users to filter any desired annotation, while others, like GEMINI has predefined annotations that restrict the user [7–10]. Command Line Interface (CLI) based tools, such as GATK-VariantFiltration, VCFtools, BCFtools filter, and Exomiser, require advanced bioinformatics and programming skills, limiting their accessibility to a broader user base [11–14]. A comprehensive comparison is provided at Table 1.

**Table 1:** A qualitative comparison between the most common VCF file filtering tools

Tool	Memory strategy	Programming language	Usability	Reproducibility	Customizability	Multi-sample VCF	Open-source
GATK-VariantFiltration	In-memory	Java	Moderate	Moderate	Moderate	Yes	Yes
BCFtools	In-memory/Disk-streaming	C	Moderate	Moderate	High	Yes	Yes
VCFtools	In-memory	C++	Moderate	Moderate	Moderate	Yes	Yes
VCF.Filter	In-memory	Perl	Moderate	Moderate	High	Yes	Yes
BrowseVCF	In-memory	Python	Easy	Moderate	High	Yes	Yes
VCF-Miner	In-memory	Java & Javascript	Easy	Moderate	High	Yes	Yes
GEMINI	In-memory/Disk-streaming	Python	Moderate	Moderate	Limited	Yes	Yes
Exomiser	In-memory	Java	Moderate	Moderate	High	Yes	Yes
123VCF	Disk-streaming	Java	Easy	Easy	High	Yes	Yes

This study aimed to develop 123VCF, a user-friendly and efficient GUI-based filtering tool that enables researchers and clinicians to define filters easily through a text file. 123VCF employs a disk-streaming real-time filtering algorithm, efficiently processing variant files without the need to load them into the computer's memory.

### Implementation

Effective variant filtering is a pivotal stage in Next-Generation Sequencing (NGS) data analysis, involving variant annotation and subsequent filtering based on user-defined criteria. However, traditional variant filtering tools often suffer from memory-intensive processes, especially when dealing with extensive datasets, as they load the entire input VCF file into memory before applying filters [13]. To address this challenge, we introduce 123VCF, an innovative tool that employs a memory-efficient algorithm for variant filtering, eliminating the need to load the input VCF file into memory. This breakthrough not only ensures faster processing but also enables seamless handling of large datasets.

123VCF is a freely available, versatile, and cross-platform tool developed using Java Swing, and it is distributed under the MIT license. The tool provides users with a user-friendly graphical interface enabling them to filter VCF files based on annotations within the "INFO" and "FORMAT" fields. Additionally, researchers can easily isolate de novo variants in multi-sample VCF files by specifying genotypes for each sample. To ensure simplicity and independence from third-party codes, all components of 123VCF were entirely developed by the authors, resulting in a straightforward and lightweight tool.

**Fig. 1:**
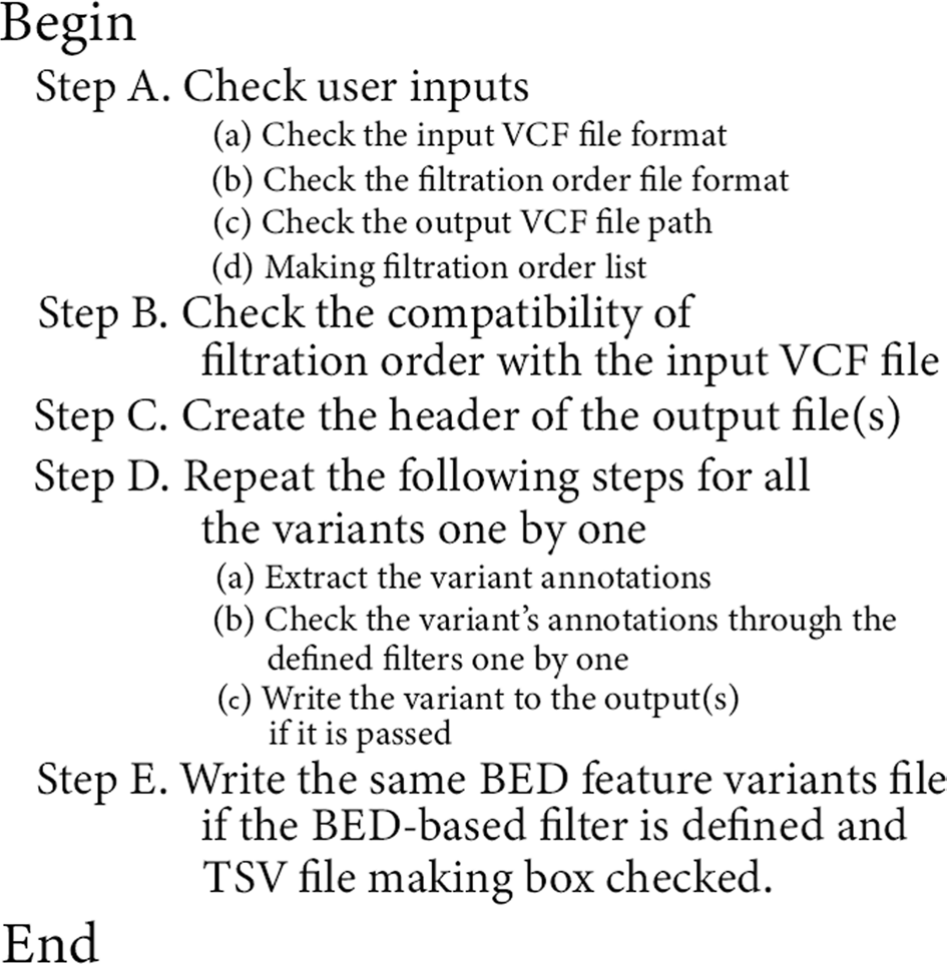
123VCF algorithm's steps

The filtering process is initiated by conducting an analysis of the filtering order file in comparison to the header section of the submitted VCF file, ensuring a comprehensive evaluation. Subsequently, each filter is systematically applied to every variant, employing intricate regular expressions rules tailored for string and numerical based filters. Through this advanced approach, only those variants that successfully meet all specified criteria, both in terms of string matching and numerical operations, are selected and documented in the designated output file(s). The underlying algorithm's core concept is visualized in Fig. 1, providing a clear representation of the methodology employed by 123VCF for efficient variant filtering. With its ease of use and powerful filtering capabilities, 123VCF emerges as a valuable tool for researchers and bioinformaticians in diverse genomic analyses.

123VCF offers users the flexibility to include or exclude heterozygous and homozygous variants from the sample, allowing for precise and customized filtering. The tool can generate a Tab-Separated Values (TSV) file containing all passed variants, which can be easily imported into spreadsheet-based programs for further analysis. Additionally, 123VCF can generate another TSV file specifically for variants that overlap with a user-provided BED file, allowing researchers and clinicians to identify possible compound heterozygous variants. These TSV files provide a convenient and customizable way to prioritize and analyze variants of interest. The efficiency of 123VCF were evaluated using a set of variant files and also compared to the most similar algorithms, demonstrating its ability to handle large datasets without compromising performance. The tool's disk-streaming real-time filtering algorithm was found to be efficient, providing accurate filtering results in a short amount of time.

123VCF provides a robust functionality that allows users to define and apply custom filtration orders using plain text files, as outlined in the user manual. This feature offers a high level of convenience, enabling users to utilize their laboratory-specific filters repeatedly without limitations. By incorporating this feature, users can streamline their workflow and enhance reproducibility, ultimately improving the efficiency and accuracy of their analysis. Furthermore, to facilitate the use of this feature, we have provided several filtering order files along with the tool, providing users with a starting point for customizing their own filtering orders.

## Results

In order to demonstrate the efficacy of 123VCF, a thorough benchmark analysis was conducted using a diverse collection of VCF files from prominent projects [10, 15–17]. To ensure consistency in annotations, ANNOVAR with identical databases was employed for all six VCF files [5]. The benchmark comprised VCF files with varying numbers of variants and samples, and the condensed results are presented in Table 2, providing information on variant and sample counts, annotated VCF file sizes, applied filters, and run time of 123VCF, BCFtools filter and GATK VariantFiltration in seconds.

**Table 2:** The benchmark results of filtering six well-known VCF files utilizing five different predefined sets of filters

File name	#Variants	#Samples	#Size of files	Number of deployed filters (Only info field filters included)				Previous filters plus GT filter	BCFtools filter runtimes	GATK Variant-Filtration runtimes
				**1** numeric filter	**3** numeric filters	**3** numeric + **3** Text-based filters	**6** numeric + **3** Text-based filters	**10** filters	**10** filters	**10** filters
HG00098.vcf	46,065	1	73.4 MB	**1.87 s**	**1.22 s**	**0.81 s**	**0.83 s**	**0.81 s**	**0.71 s**	**3.32 s**
NA12878.trio.vcf	74,362	3	324 MB	**26.14 s**	**13.71 s**	**6.94 s**	**5.57 s**	**4.10 s**	**1.25 s**	**6.87 s**
quartet.variants.annotated.vcf	300,035	4	569 MB	**34.05 s**	**25.51 s**	**7.80 s**	**7.09 s**	**6.62 s**	**2.49 s**	**12.04 s**
1 KG.chr22.anno.10kLines.vcf ^*^	9981	629	201 MB	**0.98 s**	**0.92 s**	**0.87 s**	**0.90 s**	**1.05 s**	**0.90 s**	**3.86 s**
1 KG.chr22.anno.20kLines.vcf ^*^	19,875	629	410 MB	**1.92 s**	**1.85 s**	**1.77 s**	**1.93 s**	**2.05 s**	**1.71 s**	**3.95 s**
1 KG.chr22.anno.vcf ^*^	346,660	629	6830 MB	**32.53 s**	**29.72 s**	**25.10 s**	**24.23 s**	**24.71 s**	**30.72 s**	**28.16 s**

* In these samples, the option to create the TSV file in 123VCF has been disabled owing to a cautionary notification that surfaces when the input VCF file contains over 50 samples

Additionally, the last columns demonstrate the runtimes when applying the last set of filters to the files using BCFtools filter and GATK VariantFiltration. The entire analysis was performed on a Linux Ubuntu 22.04.3 LTS operating system, equipped with an 11th Gen Intel(R) Core(TM) i7-11800H processor, 64 GB RAM, and a 512 GB NVMe PCIe Gen3 storage drive.

Table 2 clearly shows that 123VCF is an expeditious and effective filtering tool capable of processing large VCF files within seconds. The algorithm of 123VCF demonstrated precision in filtering variants in large VCF files while maintaining optimal performance, providing a significant tool for variant analysis to researchers and clinicians. It is crucial to highlight that 123VCF adopts a distinct filtering strategy compared to other available tools, making direct comparisons challenging. Nevertheless, our rigorous benchmark analysis demonstrates that 123VCF is an exceptionally efficient tool, particularly when multiple impactful filters are employed. In this benchmark, we chose to compare 123VCF with the most similar algorithms, BCFtools filter and GATK VariantFiltration tools. The runtimes of the similar tools are included in the rightmost columns of Table 2. It is important to highlight that we utilized identical uncompressed non-indexed VCF files for this benchmark.

A notable factor affecting 123VCF's performance is the I/O speed of the hard disks. Utilizing Solid-State Drives (SSD) hard drives can significantly enhance its efficiency. To optimize runtimes, we introduced an option to remove filtered-out variants from the output files, as organizing variants in the output files was identified as the most time-intensive operation in our algorithm. Additionally, 123VCF's ability to handle varying file sizes with little impact on performance makes it an invaluable resource for researchers dealing with different scales of data in NGS data analysis.

## Conclusion

In conclusion, the development of 123VCF has yielded a highly efficient VCF file filtering tool with notable advantages over existing filtering tools. The tool's versatility in allowing users to define filters based on any desired annotation, and its filtering algorithm contribute to its efficacy in genetic analysis.

Another significant advantage of 123VCF is its standalone architecture, which allows users to run the tool on a local computer without requiring an internet connection. This ensures the privacy of submitted information, making it a highly secure tool for genetic analysis.

In addition, we added a command line interface to 123VCF to make it even more user-friendly and reproducible. This will allow users to easily automate their analyses and integrate 123VCF into their existing workflows. We believe that this new feature will further increase the accessibility of 123VCF and streamline the analysis process. Our team is dedicated to providing the best possible user experience, and we are excited to continue innovating and improving the tool in the future.

## Availability and requirements

Project name: 123VCF.

Project home page: https://project123vcf.sourceforge.io.

Operating system(s): Platform independent.

 Programming language: Java. Other requirements: Java 1.8.

License: MIT. 

Any restrictions to use by non-academics: None.

## Data Availability

The compressed annotated VCF files utilized in our benchmark analysis are accessible through the project Source Forge page: https://sourceforge.net/projects/project123vcf/files/Benchmark_Data/.

## References

[1] Schutz S, Monod-Broca C, Bourneuf L, Marijon P, Montier T (2022). Cutevariant: a standalone GUI-based desktop application to explore genetic variations from an annotated VCF file. Bioinform Adv.

[2] Eidi M, Garshasbi M (2019). A novel ISCA2 variant responsible for an early-onset neurodegenerative mitochondrial disorder: a case report of multiple mitochondrial dysfunctions syndrome 4. BMC Neurol.

[3] McLaren W, Gil L, Hunt SE, Riat HS, Ritchie GRS, Thormann A (2016). The ensembl variant effect predictor. Genome Biol.

[4] Yang H, Wang K (2015). Genomic variant annotation and prioritization with ANNOVAR and wANNOVAR. Nat Protoc.

[5] Wang K, Li M, Hakonarson H (2010). ANNOVAR: Functional annotation of genetic variants from high-throughput sequencing data. Nucleic Acids Res.

[6] Cingolani P, Platts A, Wang LL, Coon M, Nguyen T, Wang L, Land SJ, Lu X, Ruden DM (2012). A program for annotating and predicting the effects of single nucleotide polymorphisms, SnpEff: SNPs in the genome of Drosophila melanogaster strain w1118; iso-2; iso-3. Fly.

[7] Salatino S, Ramraj V (2017). BrowseVCF: a web-based application and workflow to quickly prioritize disease-causative variants in VCF files. Brief Bioinform.

[8] Müller H, Jimenez-Heredia R, Krolo A, Hirschmugl T, Dmytrus J, Boztug K, Bock CVCF (2017). Filter: interactive prioritization of disease-linked genetic variants from sequencing data. Nucleic Acids Res.

[9] Paila U, Chapman BA, Kirchner R, Quinlan AR (2013). GEMINI: integrative exploration of genetic variation and genome annotations. PLoS Comput Biol.

[10] Hart SN, Duffy P, Quest DJ, Hossain A, Meiners MA, Kocher JP (2016). VCF-Miner: GUI-based application for mining variants and annotations stored in VCF files. Brief Bioinform.

[11] Smedley D, Jacobsen JOB, Jäger M, Köhler S, Holtgrewe M, Schubach M (2015). Next-generation diagnostics and disease-gene discovery with the exomiser. Nat Protoc.

[12] Danecek P, Auton A, Abecasis G, Albers CA, Banks E, DePristo MA (2011). The variant call format and VCFtools. Bioinformatics.

[13] Li H (2011). A statistical framework for SNP calling, mutation discovery, association mapping and population genetical parameter estimation from sequencing data. Bioinformatics.

[14] McKenna A, Hanna M, Banks E, Sivachenko A, Cibulskis K, Kernytsky A (2010). The genome analysis toolkit: a mapreduce framework for analyzing next-generation DNA sequencing data. Genome Res.

[15] Corpas M, Valdivia-Granda W, Torres N, Greshake B, Coletta A, Knaus A (2015). Crowdsourced direct-to-consumer genomic analysis of a family quartet. BMC Genomics.

[16] Zook JM, Catoe D, McDaniel J, Vang L, Spies N, Sidow A (2016). Extensive sequencing of seven human genomes to characterize benchmark reference materials. Sci Data.

[17] Auton A, Abecasis GR, Altshuler DM, Durbin RM, Bentley DR, Chakravarti A (2015). A global reference for human genetic variation. Nature.

